# From Fingers to Faces: Visual Semiotics and Digital Forensics

**DOI:** 10.1007/s11196-020-09766-x

**Published:** 2020-09-08

**Authors:** Massimo Leone

**Affiliations:** 1 Department of Philosophy and Educational Studies, University of Turin, Turin, Italy; 2 Department of Chinese Language and Literature, University of Shanghai, Shanghai, People’s Republic of China

**Keywords:** Face, Semiotics, Forensics, Fingerprinting, Artificial intelligence

## Abstract

Identification is a primary need of societies. It is even more central in law enforcement. In the history of crime, a dialectics takes place between felonious attempts at concealing, disguising, or forging identities and societal efforts at unmasking the impostures. Semiotics offers specialistic skills at studying the signs of societal detection and identification, including those of forensics and criminology. In human history, no sign more than the face is attached a value of personal identity. Yet, modern forensics realizes that the face can mislead and, inspired by eastern models (China, Japan, India), adopts fingerprinting. In the digital era, however, fingerprinting first goes digital, then it is increasingly replaced by facial recognition. The face is back in digital AI forensics, together with a tangle of sociocultural biases. Semiotics can play a key role in studying their surreptitious influence.

## Facial Impostures

On August 3, 2019, convicted drug dealer Clauvino da Silva tried to escape the prison of Rio de Janeiro during a visit of his 19-year old daughter [76]. The Brazilian criminal sought to impersonate her by wearing a silicon mask, a wig, eyeglasses, and the teenager’s attire, swap places, and leave the detention center under false pretenses. Policemen though became suspicious about the ‘teenager’s’ strange behavior and arrested the fugitive, recording on video the moment of his undressing.^1^ Clauvino da Silva then hanged himself in a confinement jail three days later. His criminal plan would have probably succeeded, had he worn one of the resin masks printed in 3D by Realface,^2^ the Japanese company created by Osamu Kitagawa [87]. The same kind of mask would have served the purposes also of French-Israeli citizen Gilbert Chikli, nicknamed by French policemen “the king of fraud”, who, in summer 2015, convinced several donors from around the world to transfer to him enormous sums of money; he did so through impersonating, this time through a latex mask, the then French Minister of Defense Jean-Yves Le Drian, claiming the necessity to finance the French government’s fight against terrorism [31].^3^

The face is both biologically and culturally a compelling sign of identity (or rather, a matrix of signs) [13]. Members of the human species show their faces in order to be distinguished from others and, at the same time, observe others’ faces so as to determine their identity [16]. Natural evolution has selected as adaptive both the human genetic characteristic of having a face that somatically looks different from any other and the human neurophysiological ability to tell faces apart and recognize them [97]. Some faces look more similar than others (as it happens with monozygotic twins, for instance), and some individuals might genetically be more apt than others at distinguishing and recognizing faces, yet these parts of the body have been essential signs of human personal identity for most human history, at least until Frenchman Alphonse Bertillon introduced forensic anthropometry in 1883 [8].^4^ Even in the “Bertillonage”, though, as the method was commonly called, measurements of head length (crown to forehead), head width (temple to temple), width of cheeks, and “lengths” of the right ear remained essential [9]; moreover, the same Bertillon introduced mugshots so as to assist, through the new medium of photography [38, 72], the identification of individuals [10].

## Facial Identities

Semiotics is the discipline that studies everything that can be used to lie, as Umberto Eco, one of the founding fathers of the discipline, wittily defined it [33]. Lies are also a central issue in forensics, which precisely seeks to detect and uncover the truth behind those lies that are used to organize, commit, and conceal crimes.^5^ Although the face is a ‘natural sign’, it can also be used to mislead [35]: individuals, for instance, can seek not to show their faces in the preparation or in the perpetration of a crime; they can cover them; they can wear a mask or adopt a false countenance; they can impersonate other people by ‘donning’ alternative faces (or rather, a simulacrum of them). But faces can lie also when they are not concealed, covered, masked, or made up, for, as it has been underlined at least since Augustine on [49], facial expressions themselves can be displayed so as to lie about one’s cognitive, emotional, or pragmatic status [37].

As specifically regards the face, then, a long-period dialectics takes place between the criminal possibility to lie about or through the face and the societal effort to detect and unmask such felonious lies. Representing the face first through a visual bi- (drawing, painting, engraving, photograph), then three- (sculpture), and now moving (film, hologram) artefact has a long history, for facial representations are found also in prehistorical sites [6]. Yet, the idea of using the verbal or visual representation of an individual’s face in order to identify it is relatively more recent. In western history, only divinized Roman Emperors could have their effigies represented on coins when they were still alive, and that was not meant to help their identification but, on the contrary, the identification and validation of coins themselves [15]. One of the first instances of identification of lay people through the description of their faces is the late 14th-century *Libro del Pellegrino* [“pilgrim’s book”], kept in the medieval pilgrims’ hospital of Santa Maria della Scala, Siena [78]. Here pilgrims coming from the four corners of Europe and bound to Rome used to deposit their values, together with a description of their persona that also included a verbal representation of their countenance. The systematic adoption of visual representations of faces as identification means started with photography, whose invention slightly predates the Bertillon method and, as it was mentioned earlier, was turned by French criminologist himself into an anthropometric device (the invention of mugshots) [1].^6^

## From Faces to Fingers

These, however, were soon replaced by fingerprints as more effective signs for the identification of individuals and potential criminals.^7^ Emperor Qin Shi Huangdi^8^ first used clay finger prints to seal documents (wooden tablets and whittled pieces of bamboo stringed together) [2: 15, 28]. In 1975, moreover, during excavations in Shuihudi or “tiger sleeping land”, i.e., former Chu state’s Yun-Meng (“dream in the clouds”) County in China, anonymous bamboo slips were found containing a *Ri Shu* (a county magistrate’s arbitration and litigation book) recording a Qin dynasty trial (300 BC) during which handprints and knee marks were presented as evidence [55]. That is probably the first recorded mention of handprints in a forensic setting.^9^ The custom of leaving fingerprints on a contract is attested, moreover, in the Tang period, in three borrowing acts, two of them dating from 782, the third from 786. In the three of them, the formule “畫指爲記” [Huà zhǐ wèi jì] is found, meaning: “they impressed their fingerprints as a mark” [11: 491].

The 14th-Century Persian book *Jāmiʿ al*-*tawārīkh*,^10^ attributed to Rashīd al-Dīn Ṭabīb (Persian:  ), also known as Rashīd al-Dīn Faḍlullāh Hamadānī ( ),^11^ also refers to the Chinese practice of identifying individuals from their fingerprints:

They take the fingerprints of the persons that are questioned. And the meaning of fingerprint is as follows. It has been discovered and confirmed by experience that the finger joints of all people are different. And so whenever they take a deposition from anyone, they place the paper between his fingers and on the back of the document mark the place where his finger joints touched, so that should he at some time deny his statement they can confront him with the marks of his fingers, and since these are correct, he can no longer deny it.^12^

[85: 280-1]

Western history ‘discovered’ fingerprints much later. Nehemiah Grew^13^ first published observations about the friction ridge in the *Philosophical Transactions of the Royal Society of London* in 1684. In his “Description and Use of the Pores in the Skin of the Hands and Feet” he wrote about something that the East had long discovered and used:

[…] or if any one [sic] will but take the pains, with an indifferent Glass, to survey the Palm of his Hand very well washed with a Ball; he may perceive (besides those great Lines to which some men have given Names, and those of middle size call’d the Grain of the skin) innumerable little Ridges, of equal bigness and distance, and everywhere running parallel with one another.

[50: 566]

The approach, however, was different: whereas ancient China realized that fingerprints were unique for the sake of their forensic purposes, early modern Europe still ignored the utility of the discovery but exclusively enquired about the nature of its causes [48].

Dutch anatomist Govard Bidloo^14^ followed in his 1685 book *Anatomia humani corporis*, with description of the papillary ridge (Table 14, Figure 4) [12]; sublime engravings by Gerard De Lairesse,^15^ a pupil of Rembrandt, were instrumental in visually render the anatomic discovery.^16^ Marcello Malpighi,^17^ anatomy professor at the University of Bologna, identified fingerprint ridges, spirals, and loops in his 1686 treatise *De Externo Tactus Organo*. Johann Christoph Andreas Mayer^18^ pointed out the uniqueness of fingerprints in his 1788 *Anatomische Kupfertafeln nebst dazu gehörigen Erklärungen*,^19^ yet he continued to stress their similarity. The identificatory potential of fingerprints was overlooked also by Czech anatomist Johannes Evangelista Purkinje^20^ in his *Commentatio de examine physiologico organi visus et systematis cutanei*.^21^

Several causes might have led to such negligence. First, although fingerprints were studied by anatomists, and were potentially under the eyes of everyone, they were mostly overlooked. Before the invention of the microscope and the development of modern anatomy, even an artist like Dürer, maniacally attentive to the shape of the human body, would omit representing them properly.^22^ In modern anatomy too, however, scholars long failed to notice the uniqueness of fingerprints and never suspected that they could be used for identification purposes. That was the case not only because they would adopt an anatomic rather than an anthropometric perspective, but also because of semiotic reasons. Indeed, the task of identifying individuality was demanded for centuries to another ‘sign’, that is, the face. The connection between this part of the body and the both natural and cultural drive to use it as a primary visual source of identity was so strong that it overshadowed any other semiotic means. As late as 1905, *The Lancet* would indicate that

Recognition by memory of the prisoner by someone present at his previous trial and conviction is the usual means employed at the present time of proving previous convictions in court. Some police officers, no doubt, have good memories for faces.^23^

As the founding father of US semiotics Charles S.S. Peirce would have pointed out at the turn of the 19th century, however, representations of the face and representations of fingerprints do not semiotically work in the same way [86]. Before the invention of photography, facial representations were mostly icons, that is, signs representing their objects through a relation of similarity. In drawings, paintings, and engravings, however, the symbolical dimension (in Peirce, the dimension of conventionality) was never completely absent: the artist’s style, for instance, would influence the depiction. There were some predominantly indexical facial representations too (that is, representations ‘motivated’ by a ‘physical’ link between the object and the sign), but they were rare and seldom used for identification purposes: funerary masks molded after the faces of deceased people, for instance [64]. Other facial images of this kind would be mythical, like ‘acheiropoieta’ images of the face of Jesus [62]. The task of identifying people was, therefore, demanded to other signs, such as seals.^24^ Seals, indeed, are predominantly indexical signs, meaning that their objects, the seal’s bearer, is in temporal and spatial contiguity with them. Upon the invention of photography, facial representations started to work as ‘visual seals’. Analogic photographs were the causal result of the camera’s exposition to a certain configuration of light.

Although anatomists overlooked for a long time the uniqueness of fingerprints and their potentiality as identity markers, two episodes, outside of the domain of anatomy, drew attention on it: the first, on the untrustworthiness of face photographs for legal purposes; the second, on the anthropometric usefulness of fingerprints. In 1903, a man by the name of Will West was detained in the penitentiary of Leavenworth, Kansas [22, 83]. Authorities, however, found out that a prisoner by the name of William West was already in the detention center, looked quite similar to the new inmate, and had almost the same Bertillon measurements. The episode discredited such anthropometric method, which had been in use for over one century around the world, revealing that its measurements were too vague to discriminate among individuals especially in times of generalized surveillance and mass detention. At the same time, the episode also exposed the untrustworthiness of mugshots: the photographic device and the format were indeed ‘standardizing’ facial pictures, thus decreasing their usefulness for identity detection and discrimination.^25^ Later on, it would have been found that human beings are also generally more apt at distinguishing among faces within their own ethic groups than outside of it [90]: US Caucasian policemen would probably have a hard time detecting slight differences among pictures of African-American individuals. Racism would then turn incapacity of perceptual discrimination into leanings to ethnic discrimination.

The second episode, at the opposite corner of the world, led to the conclusion that, whereas cameras would be complicated and mostly unreliable devices for the productions of indexical representations of faces (also because these representations were mostly received as iconic signs, that is, by virtue of their supposed resemblance to their objects), fingerprints could be made significant through a much less complicated and reliable technology. The first modern instance of it emerged in British colonial India, when Sir William James Herschel,^26^ Chief Magistrate of the Hooghly District in Jungipoor, India, had the fingerprints of Rajyadhar Konai, a local businessman, impressed as signs of personal identification on a contract in 1858.^27^ The same procedure was then adopted for native contracts in the following fifty-seven years, initially with full prints of right palms, then reduced to fingerprints of right middle fingers. From the semiotic perspective, the first fingerprints ever used for legal purposes would therefore add extra semiotic value not to photographs but to signatures. Indeed, from the semiotic point of view, both were icons working as indexes by virtue of their resemblance to a prototype, the difference yet being that the prototype of a signature is a conventional index traced with a writing instrument, whereas the prototype of a fingerprint is the finger itself, that is, a ‘natural’ limb.

In the following years, techniques and technologies for the production and observation of these signs improved considerably. Their purpose was to better the production of fingerprints’ representations in size, definition, and reliability, as well as the human capacity to analyze them. In 1863, French Professor Paul-Jean Coulier,^28^ of Val-de-Grâce in Paris, first observed that (latent) fingerprints could be developed on paper by iodine fuming [74]. He also suggested using a magnifying glass for observing them in relation to potential crime suspects. Subsequently, many other scholars contributed to further improvements. American microscopist Thomas Taylor’s^29^ 1877 lecture on the use of microscopes for the observation of hand marks was saluted by *The American Journal of Microscopy and Popular Science* as a “new system of palmistry”.

Hand Marks Under the Microscope. In a recent lecture, Mr. Thomas Taylor, microscopist to the Department of Agriculture, Washington, DC, exhibited on a screen a view of the markings on the palms of the hands and the tips of the fingers, and called attention to the possibility of identifying criminals, especially murderers, by comparing the marks of the hands left upon any object with impressions in wax taken from the hands of suspected persons. In the case of murderers, the marks of bloody hands would present a very favorable opportunity. This is a new system of palmistry.

(*The American Journal of Microscopy and Popular Science*,^30^ 1877, II: 89)

Such reception is interesting for it points at an intellectual development similar to that which few years earlier, in 1872, had been triggered by Darwin through his seminal essay on *The Expression of the Emotions in Man and Animals*^31^: whereas traditional physiognomy had investigated the face as a sign of personality or personal destiny, Darwin had initiated the study of facial expressions as manifestation of inner psychological states. Similarly, the difficulty to conceive palm- and fingerprints as personal identity markers was also due to the long tradition of palmistry, which had indeed focused on individual marks on people’s hands and, secondarily, fingers, but had irrationally treated them as omens.^32^ The confusion persisted in Cesare Lombroso’s study of the face, which blurred modern anthropometrics with ancient physiognomy, bestowing a new positivist aura on old superstitions.^33^

Darwin himself was acquainted with the importance of fingerprints. In 1880, he received a proposal for a classification of fingerprints by Dr Henry Faulds,^34^ the British Surgeon-Superintendent of Tsukiji Hospital in Tokyo, Japan, who, again, had realized the importance of these identity marks through his contact with a non-European visual culture, and in particular upon noticing finger marks on specimens of ‘prehistoric’ pottery:

In looking over some specimens of ‘prehistoric’ pottery found in Japan I was led, about a year ago, to give some attention to the character of certain finger-marks which had been made on them while the clay was still soft.

[39: 605].^35^

Darwin was too old and ill to personally study the matter, but he realized its importance, and forwarded the dossier to his cousin, Francis Galton.^36^ Galton then became a pivotal figure in the history of fingerprint identification. Fauld also first proposed the use of printer ink for obtaining fingerprints and first identified a greasy fingerprint left on an alcohol bottle. The birthdate of the modern forensic use of fingerprints is, however, 1891, when Juan Vucetich,^37^ an Argentinian Police Official, started to collect fingerprint files based on Galton pattern types [47]. The first criminal fingerprint identification also took place in Buenos Aires, Argentina, in 1892, when Inspector Eduardo Alvarez, trained by Vucetich, identified through fingerprints on a door post Francisca Rojas, a woman who had murdered her two sons and simulated to cut her own throat so as to exculpate herself. Her bloody print was left on a door post, proving her identity as the murderer. When, on August 21, 1911, Italian artist Vincenzo Peruggia^38^ stole the Mona Lisa from the Salon Carré of the Louvre, the police arrested two young men who would subsequently become world famous, Guillaume Apollinaire and Pablo Picasso. Alphonse Bertillon was among those who interrogated the young Picasso at the Palais de Justice and was able to prove his innocence by comparing his left fingerprint with the one impressed by the thief on the glass that would shield the stolen painting in the Louvre.

By the early twentieth century, then, fingerprints started to become a mainstream forensic sign. In 1900, the United Kingdom Home Secretary Office promoted an inquiry into “Identification of Criminals by Measurement and Fingerprints” and recommended replacing the Bertillon system with fingerprinting, mainly relying on Edward Richard Henry’s^39^ book *The Classification and Use of Fingerprints* (1900) [53]. The system proposed therein was then officially adopted by the Fingerprint Branch at New Scotland Yard (Metropolitan Police) in July 1901 [4].

An important step in the semiotic history of fingerprints took place in 1914, when Hakon Jørgensen, from the Copenhagen Denmark Police, lectured at the International Police Conference in Monaco about the possibility of sending fingerprints through telegraphic communication. Fingerprints were indexically used icons. They were therefore analogic artefacts, whose shape would be created through physical contiguity with finger ridges and would signify through pattern resemblance with them. Jørgensen first proposed translating such analogic signs into digital ones through the binary code of the Morse alphabet. An English description of the method was first published in Copenhagen in 1922 under the title *Distant Identification* (after the Danish original of 1916):

Let us presume that a person arrested in Stockholm, is identical with a sought-for burglar from Paris. The description of the arrested person tallies with that of the one wanted, but the arrested person denies the identity and might also possibly carry such credentials as show him to be identical with a French merchant travelling on business. The police suspect him immediately of carrying false credentials. How can the Stockholm police decide this case?

[57: 4].

By 2012, INTERPOL’s Automated Fingerprint Identification System repository contained more than 150,000 sets of fingerprints for important international criminal records from 190 member countries. Currently, in 2020, the US Department of Homeland Security’s Office of Biometric Identity Management (OBIM), contains over 120 million persons’ fingerprints. “Fast capture” technology presently enables recording of ten simultaneous fingerprint impressions in circa 15 s per person. India’s Unique Identification project, also known as Aadhaar, is currently the largest digital database of fingerprint impressions in the world, with 1.11 billion Aadhaar numbers as of January 2017.

## From Fingers to Faces

The electronic fingerprint recognition feature known as “Touch ID” has been sold by Apple as part of all iPhones since 2013’s iPhone 5S up until 2017’s iPhone 8 and 8 Plus; it has been on all iPads since 2014’s iPad Air 2 except for 2018’s iPad Pro (3^rd^ generation). In 2015, Apple introduced a faster second-generation Touch ID in the iPhone 6S; a year later, in 2016, it was also integrated in the MacBook Pro on the right side of the Touch Bar and in the 2018 MacBook Air. The identification of human beings through indexical and iconic impressions of their finger ridges was developed as a way for societal law enforcement agencies to single out citizens and above all potential criminals. The digitalization of this anthropometric technique, though, led to its miniaturization and integration into portable personal devices such as smartphones, tablets, and computers. In the frame of the history of communication, this process entails that these devices and their data become the exclusive ‘territory’ that fingerprints are meant to protect [65]. There is a reverse of the medal, though: whilst users protect their devices and data through impressions of their bodies, these devices have potential access to the same signs and identity marks that they, the users, present so as to be identified by state apparatuses, for instance at frontier controls around the world.

There is, moreover, a critical difference between passing a frontier control by demonstrating one’s identity through fingerprints and unlocking a smartphone with TouchID. Whereas in the first case, a whole system of human and machinic surveillance makes sure that the impressions are actually indexically connected to a living body, in the second case, devices only presuppose that the fingerprint actually belongs to a finger. In September 2013, the biometrics hacking team of the Chaos Computer Club (CCC) successfully bypassed the biometric security of Apple’s Touch ID. A fingerprint of the phone user, photographed from a glass surface, was used to create a fake finger that could unlock an iPhone 5s secured with Touch ID. In practice, CCC used the same method that, in detective stories, is adopted to inculpate someone by leaving his or her fingerprint impressions on the crime scene. Frank Rosengart, CCC spokesperson, concluded:

The fingerprint as security feature loses more and more of his value the more biometric verification systems use it as a feature. The same fingerprint, which is scanned in high resolution at the grocery store shall be used at the border for verification. No customer can verify if the high-resolution fingerprint is stored anyway.^40^

The adoption of biometric security procedures for personal and portable devices cannot be understood in purely technical terms. As these devices become the repository of an increasing amount of personal and sensitive data, their security turns into a value but also into a matter of competition among global Hi-Tech companies. Securing access to a smartphone through digital fingerprints, then, bestows on such portable communication technology the aura of state security measures, such as, indeed, the finger ridge impressions adopted at frontier controls and in penitentiaries.

Significantly, though, Apple announced Face ID during the unveiling of the iPhone X on September 12, 2017. Face ID was meant to replace Touch ID on iPhone (X, XR, XS, XS Max, 11, 11 Pro, 11 Pro Max) and iPad Pro (third generation). On the one hand, the passage from fingerprints ID to face ID seems to reverse the tendency from facial to fingerprints identification, which has been underlined earlier as characterizing the history of western forensics. On the other hand, this reversal too can be fully understood only within a semiotic framework. In the history of human cultures, there is no comparison between the semiotic aura of the face and that of fingerprints. At least in the west, humans have realized only after a long and tortuous development that fingerprints could be used to single out individuals. Such realization was reached upon the invention of the microscope and the institution of modern anatomy, accompanied by the development of modern engravings and, subsequently, photography.

On the contrary, the face has been considered as a prime marker of individual identity since the birth of humanity. As it has been already underlined, the capacity of using the face to communicate personal identity is probably part of the biology of the human nature. Hence, by reproducing this capacity through the artificial intelligence of a smartphone, Face ID turns the relation between users and their iPhones into an even more personal ones: iPhones are no longer like a frontier control agent, identifying people through their fingerprints, but like a friend, recognizing users from their faces.

The internal semiotics of this apparently friendly recognition should be taken into account too. Face ID does not recognize faces by comparing their present visual appearance with a past visual appearance of theirs stored in the device’s memory through a digital representation; rather, it recognizes them as a blind person would recognize a friend’s face, that is, through ‘touching it’ and producing, then, a digital ‘mold’, a sort of invisible funerary mask of the face. Indeed, the Face ID hardware consists of a sensor with three modules: a dot projector that projects a grid of small infrared dots onto the user’s face, a module, the flood illuminator, that reads the resulting pattern and generates a 3D facial map, and an infrared camera that takes an infrared picture of the user. It is disquietingly called the “true depth camera system”.

## Toward a Semiotics of Digital Forensics

Depending on whether predominantly symbolic (like a password), iconic (like a picture), or indexical (like a fingerprint) means are used to protect or detect the personal identity of individuals, the dialectics between the secrecy of the citizen and the intrusiveness of society changes. Finger- and facial prints might seem more personal and private, avoiding users the task of choosing, remembering, periodically changing, and also potentially forgetting a password; as a consequence, they generate an imaginary of proximity, efficacy, and even naturalness, which is particularly compelling in the case of the face; users activate their phones by simply looking at them, exactly as they would draw the attention of a human interlocutor. As biometrics hackers have emphasized, though, icons and especially indexes cannot be replaced, for they are not arbitrary like passwords but motivated like fingerprints. The only way to change the biometric value of fingerprints is to modify their object, that is, fingerprints themselves (which is what criminals or fugitives often do, erasing or at least blurring their finger ridges with acid or other means) [93].

The result of adopting biometrics as access code to personal devices is, hence, that whoever somehow gets hold of the body part (a replica of the finger, a replica of the face) has permanent and complete access to all security systems that adopt the same object, including national frontier controls. That plays a role also in the relation between the citizen and law enforcement agencies. There is, indeed, the possibility that users might be forced to unlock their phones by someone (a criminal, but also a policeman or FBI investigator) simply pointing the phone at their, the users’, faces. Simply closing one’s eyes would block the unlocking attempt, for Apple Face ID requires eye contact, yet it remains that, in many jurisdictions, symbolical passcodes like passwords offer more privacy rights than indexical passcodes, exactly because the former constitute a mental content, whereas the latter are a bodily display. Under the US Fifth Amendment, for instance, passwords are a piece of testimonial evidence, so that a judge cannot force a suspect to disclose them unless the nature of the content that will be thus disclosed is already reasonably known, whereas body parts like fingerprints and even more facial prints, whose use as passcodes is more recent, do not undergo the same protection.

Such preoccupation about the testimonial or non-testimonial nature of facial evidence also emerges in connection with technology, techniques, and devices of face detection. As it has been pointed out earlier, faces are a formidable marker of personal identity. It is, therefore, straightforward that facial evidence is treated as key in investigations that societies carry on, through their law enforcement agencies, about crimes and their perpetrators. Gathering facial evidence about a criminal in the preparation or in the perpetration of a felony is of primary investigative value. That is the rationale behind the creation and development of several forensic techniques and technologies, as well as criminologist theories.

Forensic ‘art’, that is, ‘art’ used in law enforcement or legal proceedings in order to gather visual evidence about a crime, is often concerned with facial representations. Composite drawing, crime scene sketching, image modification and identification, courtroom drawings, demonstrative evidence, and postmortem body reconstruction all might involve facial approximations of some sort. That is particularly evident in composite drawings, whose central purpose is to help investigators gather visual clues based on verbal descriptions by victims and other witnesses about the physical appearance, and especially the face, of potential criminals. From the semiotic point of view, composite drawing involves inter-semiotic translation, that is, translation from an essentially verbal language into an essentially visual one. During investigations, ideally in the 48 hours after the crime, witnesses verbally describe to investigators the facial appearance of the suspects, often helped by the questions of forensic artists. These, in turn, must convert the received verbal clues into visual forms, composing a sketch of the suspect’s face that might be used as visual lead for investigation.

Despite the evolution of visual and graphic technology, hand drawing is still the preferred method of forensic art by many law enforcement agencies, including FBI [92]. Artificial Intelligence is indeed increasingly trained at being proficient in recognizing objects in images, and in describing their content through verbal language, yet the opposite is still problematic: algorithms passing from a verbal description of a face to the depiction of its countenance are still underdeveloped. A first mechanical system for the production of facial composites, the “Identikit”, was introduced in the US in 1959; it consisted of drawings of facial features on transparent acetate sheets that could be superimposed on one another to produce the composite image. In 1970, a system called “Photofit” was introduced, aiming at more realistic composites through using photographs of facial features.

The specific cognitive nature of the inter-semiotic translation between the verbal description of a face and the visual representation of it makes the transition from human, manual composite drawing to photographic composite and, even more, to AI composites, particularly hard. Indeed, the task of the forensic ‘artist’ is not to represent, through hand-drawing or by composing fragments of drawings, photographs, or images of other kinds, a face whose countenance is known and deposited as mental depiction in the visual memory of the describing individual, albeit not visible anywhere. In this case, the inter-semiotic translation would be necessary so that individuals, others than the describing subject, and namely law enforcement officers, might see the face that the subject remembers, or at least a resembling representation of it. The situation is, in fact, different: subjects who have been victims of or witnesses to a crime do not remember the face of the criminal as one would remember the face of a movie star seen on a picture; they rather remember it as a fleeting foreshadow of a face, with a degree of mental precision that is affected by several factors: (1) the innate cognitive ability of the victim at remembering faces: some individuals are genetically more apt than others at detecting, retaining, identifying, and recognizing faces; some victims or witnesses, instead, might even be affected by prosopagnosia, or ‘face blindness’, a cognitive disorder of the ability to recognize familiar faces, including one’s own; (2) the particular somatic countenance of the remembered face; the psychology of face perception has accumulated much evidence about how certain natural or cultural features of the face are more likely to be retained in the memorization of a face; already Bertillon would, for instance, underline the importance of the nose in face identification and recognition; (3) the linguistic ability of the victim or witness at describing reality, and particularly faces, through verbal language; (4) the contextual circumstances of the perception, which can render the memorization of the face particularly problematic, because of the exceedingly rapid or partial exposition of the victim or witness to the face (in many cases, criminals will seek to hide their faces through sunglasses, helmets, masks, facial hair, etc.) and because of the traumatic conditions in which such exposition takes place; (5) the cognitive and cultural biases through which perception and memorization unfold, including racist prejudice concerning the relation between ethnicities and crime.

During the composite drawing, identikit, or other more recent techniques, therefore, the victims or witnesses must not only describe to the forensic ‘artist’ the face that they have seen, but simultaneously (1) describe some of its features for the sake of their depiction and (2) use such depiction as visual feedback so as to test the verbal description itself and its visual rendering. In this domain too, however, artificial intelligence is becoming increasingly present [21]. Whereas in the traditional composite drawing, subjects were presented with variations of face parts, with the aim of assembling the best approximation to the suspect’s face, in evolutionary drawings, subjects are presented with images of whole faces, whose features progressively evolve towards a final result following the answers offered by the subjects themselves. This method has the advantage that subjects must not verbally describe what they seem to remember in relation to the tentative drawings, but simply answer affirmatively or negatively about the degree of resemblance between the face that they are making an effort to mentally recollect and the digital pictures that the system progressively presents to them.

Forensic ‘art’ does not include only composite drawing but also (a) image modification (including age progression and regression and clarifying of images), meant at an enhancing the existing photograph of a suspect in order to help an investigator and/or trial attorney; (b) image identification, that is, the visual representation of a person’s distinguishing features for future reference, for example, so as to identify suspects who attempt to alter their countenance in order to evade capture, or in ‘cold cases’ in which the individuals’ appearance may have changed since the criminal event; (c) crime scene sketching, through both bi- and three-dimensional rendering; (d) postmortem drawing, which consists in the professional attempt at reconstructing the way in which a deceased person might have looked, especially in cases where the body and particularly the face is overly damaged by an accident or decomposition [14]. As the digital increasingly enters the world of artists, not only in terms of available technology and ensuing techniques, but also in terms of the ‘iconic ideology’ that these technological and technical changes entail, artificial intelligence too becomes more and more present in all the domains of ‘forensic art’.

## The Rhetoric of Digital Visual Evidence

Ethical and juridical questions are likely to emerge as the digital, and particularly artificial intelligence, penetrate the domain of ‘forensic art’. Many jurisdictions would already problematize the role of the forensic ‘artist’ also in the pre-digital world, seeking to determine the relevance and impact of the artist’s intervention (and, hence, potential bias) in the construction or reconstruction of evidence. Forensic ‘sculpture’, for instance, that is, the creation of three-dimensional models reproducing some presumed somatic characteristics of either the suspect or the victim,^41^ has not been legally recognized for positive identification, since it heavily relies on the artist’s bias, and is therefore taken into account in an advisory capacity only [45].

More and more, however, machines are involved into the production, post-production, and reconstruction of digital images of faces, with an increased use of various forms of artificial intelligence in performing such tasks. The face is already a central preoccupation in the current European GDPR and in many other present-day juridical and legal frameworks. The issue of images of people’s faces being automatically detected, memorized, and recognized by machines prominently features in the public imaginary, in the mass-media, and also in the reflections of scholars in the fields of Artificial Intelligence and Law. The either testimonial or non-testimonial value of automatic facial recognition evidence, for instance, is already the object of vast jurisprudence.^42^ Yet, scholarly reflection, and even more thus legislation, are always one or several steps behind the evolution of technology and the socio-cultural challenges that it entails. The evaluation of the either testimonial or non-testimonial value of automatic facial recognition, for instance, is largely based on the idea of a humanly supervised technology. As such technology ‘improves’, however, becoming less and less dependent on human supervision, it becomes urgent to reflect in terms of where, when, and to what extent a human mental agency is introduced in the process that leads to automatic facial detection and recognition (an agency with its entire baggage of sociocultural as well as legal biases). A current trend in the development, implementation, and usage of artificial intelligence tends to move but also to rhetorically ‘conceal’ the intermission of humans away from the stage of technological usage (also in order to ‘market’ the autonomy, cost-efficiency, and impartiality of AI) towards ‘invisible’ stages of planning and training. Most convoluted neural networks for automatic facial recognition, for instance, must be trained with pictures of human faces, whose selection is largely demanded to human trainers. In the case of forensic ‘art’, moreover, the evaluation of such part of human agency should focus on the specific software that is used for the production and post-production of images, contrasting the present-day tendency to ‘naturalize’ the technology and the ensuing techniques of digital imagery.

Semiotics, meant as the discipline that seeks to understand the cultural patterns underpinning largely naturalized social processes, will have to play an essential role in uncovering the frequently invisible language through which present-day visual evidence is digitally created.

## Conclusions

Semiotics promotes the revolutionary awareness that the body is never an object but always a matrix of signs that are constantly interpreted in the interpersonal arena. These signs, in various configurations of coalescence, may vary depending on sundry aspects going from the bodily parts that a culture focuses on—encouraging or discouraging their visibility [66]—to the techniques and technologies that enable the intentional representation of the body and its social meaning. In any case, the body exists for itself and for the other bodies—as well as for the community and its institutions—not as an inert entity, but as a dynamic source of semiosis. The disrupting novelty brought about by digital technology, network communication, and especially artificial intelligence, is that this body now exists as a matrix of signs more and more not only for other human beings, but also for non-biological entities that are endowed with increasingly complex cognitive abilities, including those that turn visual stimuli into intelligence items.

Such technological watershed might also imply a cultural fracture. Representing the other human being for the purposes of recognition and identification is a very old need of humanity. In many ancient cultures, bodies are not represented only to evoke the generic idea of a body, but also and above all to stimulate the mental and communicative simulacrum of the specific and unique body of an individual. The human species, indeed, comes about with the perceptual evidence that bodies look all different, although they might be grouped along gradients of similarity and dissimilarity according to changing criteria of articulation, clustering, and categorization. This ideology of individuality, affecting both the human perception and the internal and external representations to which it gives rise, is a product of natural evolution, concentrating the production and reception of difference and identity in the face. That is why the face is such an important bio-political capital: like no other part of the human body, it allows the development of a whole series of rhetorical procedures of individualization, distinction, representation, recognition, and identification.

But the face exists beyond nature. The semiotic approach is fundamental exactly because it reveals that the bio-political capital of singularity enshrined in the face is intercepted by a complex series of discourses that bestow a culturally and historically specific significance to it. All human beings have a face, yet not all human beings have a visage that society considers as the somatic cornerstone of identity and its signification/communication. A face turned into a visage, moreover, immediately loses its neutral character and becomes a field of tension between social (cognitive, emotional, and pragmatic) instances of individualization and recognition and opposite social instances of homologation and anonymity. It is exactly in this field that, on both sides, techniques and technologies are deployed so as to increase or decrease the exposure of the visage to social processing and signification/communication. From this point of view, the veil hiding the face of a Muslim woman and the sophisticate algorithm pixelizing the digital image of a child’s face in the photograph of a crime scene are outcomes of the same ideological force, the one that tends towards the concealment of the visage as place of human singularity in the social discourse.

The contribution of semiotics to the study of these social phenomena cannot however consist uniquely in revealing the ideological energies that underpin the singularization/ostentation or, on the contrary, the banalization/occultation of the face as visage. Accrued and increasingly qualified cooperation with digital studies should lead to the awareness that the techniques and technologies of the face are no longer simply a tool, expressing an underlying ideology; their complexity and the speed of their evolution is now such that the digital tool itself becomes a source of new ideological trends; the medium is the message in the sense that what now technically allows the identification or, on the opposite, the anonymity of the face, gives shape to the predominant ideology of the visage. Monitoring through the lenses of semiotics the evolution of forensic techniques of identification means also understanding what human forces surreptitiously model the social ideologies of the visage, turning the most singular part of the visible body into a field of biopolitical tension.

## Footnotes

1 Video available at https://www.theguardian.com/world/2019/aug/05/brazil-gang-leader-dresses-up-as-teenage-daughter-in-jail-break-attempt (last accessed January 19, 2020).

2 For an overview of the company, see http://real-f.jp/en_company.html (last accessed January 19, 2020).

3 For a history of impostors, see [58, 98]; in literature, see [70]. The present essay too adopts a perspective combining cultural history and semiotics of culture; from the former, that is, from the gathering and analysis of traces and documents witnessing to the human attempts at systematically identifying individuals in a community—especially in those circumstances in which such identification mattered and was problematic (paternity, inheritance, crime, transfer of wealth, transmission of illnesses, travel, etc.)—it can be understood how local ideologies and existing techniques have decisively conditioned the forms and methods adopted to ‘signify’, ‘read’, and ‘communicate’ the identity of individuals; from the latter, that is, from the semiotic study of the signs and practices used for the identification, one can grasp continuities and discontinuities in the semiotic ideologies of identity attribution, gaining insights about the biases that underpin each semiotic paradigm of categorization of citizens and classification of their ‘signs’ in the space of the community.

4 Literature on Bertillon is extensive; see [77, 81, 82].

5 On the relation between semiotics and forensics, see [30]; cfr also [34, 91].

6 See also [18, 22, 43].

7 On the early history of fingerprints, see [24, 51, 59, 60, 79, 80].

8 秦始皇帝; Handan, 260 BC – Shaqiu 210 BC), born Yíng Zhèng (嬴政).

9 See also [3: 147, n. 117, 20, 22: 60, and 99].

10 Arabic: *Compendium of Chronicles*; Mongolian: Судрын чуулган; Persian:  .

11 1247–1318. See [52: 264, n. 8].

12 Original text in Blochet, Edgar, ed. 1911. *Djami El-tévarikh; Histoire générale du monde* (E.J.W. Gibb Memorial Series, 18). Leyden: E.J. Brill.

13 Mancetter Parish, Warwickshire, 26 September 1641–London, 25 March 1712. See [7: 27–29]. On Grew, see also [26, 61].

14 Amsterdam, 12 March 1649–Leiden, 30 March 1713.

15 Liège, 11 September 1641–Amsterdam, 21 July 1711.

16 On the relation between Bidloo and Lairesse, see [56].

17 Crevalcore, Bologna, 10 March 1628–Rome, 29 November 1694; see [11, 46, 71].

18 Greifswald, 8 December 1747–Berlin, 5 November 1801.

19 Leipzig: Decker, 1783-1794, 6 volumes; vol. 4: “Elf Kupfertafeln von den Sinnwerkzeugen und den Brüsten”, Table II.

20 Libochovice, 17 December 1787–Prague, 28 July 1869.

21 Breslau, Prussia: University of Breslau Press, 1823; see [29].

22 Fingerprints are used as visual patterns in prehistoric ‘art’ [32], bur are seldom represented in pre-modern art. See [69, 94].

23 The Lancet 1905, 165, 4263, 13 May 1905: 1280–1281.

24 See [5, 88, 96].

25 See [17, 72].

26 Slough, UK, 9 January 1833 – Hawkhurst, UK, 24 October 1917.

27 See [54, 89].

28 Paris, 1824–1890. See [67].

29 1820–1910.

30 Published by New York, NY: Industrial Publication Company.

31 London: John Murray; see [36].

32 See [44, 63, 84].

33 See [25, 27, 95].

34 Beith, UK, 1 June 1843 – Wolstanton, UK, 19 March 1930; see [75].

35 See also [40–42].

36 Sparkbrook, UK, 16 February 1822 – Haslemere, UK, 17 January 1911.

37 20 July 1858, Hvar, Croatia – Dolores, Argentina, 25 January 1925.

38 Dumenza, Italy, 8 October 1881 – Saint-Maur-des-Fossés, France, 8 October 1925.

39 London, 26 July 1850–Ascot, 19 February 1931.

40 https://www.ccc.de/en/updates/2007/umsonst-im-supermarkt.

41 See also [68].

42 See [19, 73]; cfr [23].
